# Supplementary material for: Opportunities and challenges within green spaces during COVID-19: Perspectives of visitors and managers in Maine, USA
Source: PLoS One. 2025 Apr 22;20(4):e0320800. doi: 10.1371/journal.pone.0320800 (PMC12013927; doi:10.1371/journal.pone.0320800)
Supplement: S1 Table — Raw data for the relevant data sets from QV-21. (DOCX) [file pone.0320800.s001.docx]

| Participant ID | Traveling to rural destinations makes me feel safe during the pandemic | I am not concerned with safety when choosing to travel to destinations | Traveling to areas that have higher rates of COVID-19 vaccinations makes me feel safer | Traveling to nature-based destinations (such as a park) makes me feel safe during the pandemic | I am personally worried about COVID-19 |
| --- | --- | --- | --- | --- | --- |
| 1 | 6 | 6 | 4 | 6 | 4 |
| 2 | 4 | 2 | 4 | 4 | 4 |
| 3 | 1 | 2 | 6 | 6 | 4 |
| 4 | 6 | 2 | 4 | 6 | 3 |
| 5 | 6 | 2 | 6 | 6 | 4 |
| 6 | 4 | 4 | 4 | 4 | 3 |
| 7 | 7 | 4 | 7 | 7 | 4 |
| 8 | 4 | 2 | 4 | 6 | 4 |
| 9 | 6 | 1 | 7 | 6 | 4 |
| 10 | 4 | 2 | 4 | 6 | 4 |
| 11 | 4 | 4 | 4 | 4 | 5 |
| 12 | 7 | 2 | 1 | 6 | 4 |
| 13 | 6 | 2 | 7 | 7 | 5 |
| 14 | 6 | 1 | 6 | 6 | 4 |
| 15 | 2 | 6 | 2 | 4 | 5 |
| 16 | 4 | 4 | 4 | 4 | 3 |
| 17 | 1 | 7 | 1 | 1 | 1 |
| 18 | 6 | 2 | 6 | 6 | 4 |
| 19 | 6 | 1 | 1 | 6 | 4 |
| 20 | 6 | 4 | 6 | 6 | 4 |
| 21 | 4 | 1 | 4 | 4 | 3 |
| 22 | 6 | 1 | 6 | 6 | 4 |
| 23 | 6 | 6 | 6 | 1 | 5 |
| 24 | 6 | 4 | 4 | 6 | 2 |
| 25 | 7 | 2 | 7 | 7 | 5 |
| 26 | 6 | 4 | 4 | 6 | 4 |
| 27 | 4 | 2 | 4 | 4 | 3 |
| 28 | 4 | 1 | 6 | 6 | 4 |
| 29 | 6 | 1 | 6 | 6 | 4 |
| 30 | 2 | 2 | 2 | 2 | 3 |
| 31 | 4 | 4 | 1 | 7 | 4 |
| 32 | 4 | 2 | 4 | 6 | 5 |
| 33 | 7 | 7 | 7 | 7 | 5 |
| 34 | 6 | 2 | 6 | 6 | 4 |
| 35 | 4 | 6 | 1 | 4 | 1 |
| 36 | 7 | 2 | 7 | 7 | 4 |
| 37 | 7 | 1 | 7 | 7 | 3 |
| 38 | 7 | 2 | 6 | 7 | 2 |
| 39 | 6 | 2 | 6 | 6 | 4 |
| 40 | 7 | 7 | 7 | 7 | 5 |
| 41 | 6 | 6 | 4 | 6 | 4 |
| 42 | 6 | 6 | 4 | 4 | 3 |
| 43 | 2 | 4 | 6 | 4 | 4 |
| 44 | 4 | 6 | 1 | 6 | 4 |
| 45 | 7 | 7 | 6 | 6 | 5 |
| 46 | 4 | 4 | 4 | 4 | 3 |
| 47 | 6 | 2 | 6 | 6 | 4 |
| 48 | 4 | 6 | 7 | 6 | 4 |
| 49 | 4 | 1 | 6 | 4 | 5 |
| 50 | 6 | 6 | 6 | 6 | 4 |
| 51 | 6 | 4 | 1 | 6 | 4 |
| 52 | 4 | 2 | 4 | 4 | 4 |
| 53 | 4 | 4 | 4 | 4 | 1 |
| 54 | 6 | 4 | 7 | 6 | 5 |
| 55 | 4 | 4 | 4 | 4 | 3 |
| 56 | 6 | 2 | 6 | 6 | 4 |
| 57 | 6 | 4 | 6 | 7 | 4 |
| 58 | 7 | 1 | 7 | 7 | 5 |
| 59 | 4 | 4 | 4 | 4 | 3 |
| 60 | 4 | 1 | 1 | 6 | 4 |
| 61 | 4 | 6 | 7 | 4 | 5 |
| 62 | 6 | 1 | 7 | 7 | 5 |
| 63 | 4 | 2 | 4 | 4 | 3 |
| 64 | 6 | 7 | 4 | 6 | 3 |
| 65 | 7 | 1 | 1 | 7 | 5 |
| 66 | 4 | 2 | 2 | 4 | 3 |
| 67 | 7 | 1 | 7 | 6 | 4 |
| 68 | 6 | 6 | 6 | 6 | 4 |
| 69 | 4 | 2 | 4 | 4 | 4 |
| 70 | 2 | 1 | 1 | 1 | 5 |
| 71 | 4 | 4 | 2 | 4 | 3 |
| 72 | 7 | 2 | 4 | 7 | 3 |
| 73 | 6 | 2 | 6 | 4 | 5 |
| 74 | 7 | 2 | 2 | 7 | 2 |
| 75 | 4 | 4 | 2 | 4 | 1 |
| 76 | 7 | 7 | 7 | 7 | 2 |
| 77 | 7 | 1 | 1 | 7 | 5 |
| 78 | 4 | 6 | 4 | 4 | 3 |
| 79 | 4 | 4 | 4 | 4 | 3 |
| 80 | 6 | 4 | 6 | 6 | 4 |
| 81 | 4 | 2 | 2 | 6 | 3 |
| 82 | 4 | 2 | 4 | 6 | 4 |
| 83 | 6 | 1 | 6 | 7 | 5 |
| 84 | 7 | 6 | 4 | 2 | 3 |
| 85 | 4 | 1 | 4 | 4 | 5 |
| 86 | 7 | 2 | 4 | 7 | 3 |
| 87 | 6 | 4 | 2 | 2 | 2 |
| 88 | 7 | 6 | 6 | 6 | 4 |
| 89 | 6 | 7 | 7 | 6 | 2 |
| 90 | 6 | 2 | 6 | 6 | 5 |
| 91 | 7 | 1 | 6 | 7 | 5 |
| 92 | 2 | 1 | 6 | 2 | 5 |
| 93 | 7 | 1 | 4 | 7 | 4 |
| 94 | 4 | 4 | 4 | 4 | 5 |
| 95 | 6 | 4 | 6 | 4 | 4 |
| 96 | 7 | 6 | 7 | 7 | 5 |
| 97 | 6 | 2 | 6 | 6 | 5 |
| 98 | 6 | 1 | 2 | 6 | 5 |
| 99 | 4 | 4 | 7 | 7 | 1 |
| 100 | 7 | 1 | 4 | 7 | 4 |
| 101 | 6 | 2 | 6 | 7 | 4 |
| 102 | 7 | 1 | 6 | 7 | 4 |
| 103 | 6 | 2 | 7 | 6 | 2 |
| 104 | 6 | 4 | 7 | 6 | 5 |
| 105 | 4 | 1 | 7 | 7 | 4 |
| 106 | 6 | 1 | 6 | 7 | 4 |
| 107 | 7 | 1 | 7 | 6 | 5 |
| 108 | 6 | 6 | 6 | 6 | 5 |
| 109 | 7 | 2 | 6 | 7 | 3 |
| 110 | 4 | 6 | 6 | 2 | 1 |
| 111 | 6 | 7 | 4 | 6 | 5 |
| 112 | 6 | 1 | 1 | 6 | 5 |
| 113 | 6 | 1 | 6 | 6 | 1 |
| 114 | 4 | 6 | 4 | 6 | 2 |
| 115 | 4 | 4 | 4 | 4 | 3 |
| 116 | 6 | 2 | 4 | 6 | 5 |
| 117 | 7 | 1 | 7 | 7 | 4 |
| 118 | 4 | 6 | 6 | 4 | 4 |
| 119 | 7 | 1 | 7 | 7 | 4 |
| 120 | 2 | 7 | 7 | 4 | 5 |
| 121 | 7 | 1 | 7 | 7 | 4 |
| 122 | 4 | 1 | 7 | 6 | 4 |
| 123 | 4 | 1 | 7 | 6 | 3 |
| 124 | 2 | 2 | 6 | 6 | 5 |
| 125 | 7 | 2 | 7 | 7 | 4 |
| 126 | 7 | 2 | 7 | 7 | 3 |
| 127 | 6 | 6 | 6 | 6 | 2 |
| 128 | 6 | 6 | 6 | 6 | 4 |
| 129 | 7 | 1 | 7 | 7 | 4 |
| 130 | 6 | 7 | 6 | 7 | 5 |
| 131 | 4 | 2 | 7 | 6 | 4 |
| 132 | 7 | 2 | 1 | 6 | 1 |
| 133 | 1 | 1 | 2 | 2 | 4 |
| 134 | 4 | 6 | 6 | 4 | 5 |
| 135 | 6 | 4 | 6 | 7 | 4 |
| 136 | 4 | 6 | 4 | 4 | 4 |
| 137 | 6 | 2 | 4 | 6 | 3 |
| 138 | 4 | 4 | 4 | 4 | 4 |
| 139 | 4 | 2 | 1 | 4 | 4 |
| 140 | 6 | 2 | 6 | 7 | 4 |
| 141 | 6 | 6 | 6 | 6 | 4 |
| 142 | 1 | 2 | 7 | 4 | 1 |
| 143 | 7 | 7 | 1 | 7 | 5 |
| 144 | 1 | 1 | 1 | 1 | 4 |
| 145 | 4 | 6 | 6 | 6 | 4 |
| 146 | 6 | 2 | 6 | 6 | 5 |
| 147 | 4 | 1 | 1 | 6 | 3 |
| 148 | 7 | 2 | 1 | 7 | 4 |
| 149 | 6 | 6 | 4 | 7 | 3 |
| 150 | 6 | 6 | 6 | 6 | 5 |
| 151 | 7 | 1 | 2 | 7 | 4 |
| 152 | 6 | 6 | 6 | 6 | 3 |
| 153 | 1 | 4 | 2 | 4 | 5 |
| 154 | 7 | 7 | 7 | 7 | 5 |
| 155 | 2 | 1 | 1 | 4 | 3 |
| 156 | 4 | 4 | 1 | 4 | 1 |
| 157 | 4 | 1 | 1 | 4 | 4 |
| 158 | 6 | 6 | 4 | 6 | 3 |
| 159 | 6 | 6 | 6 | 6 | 5 |
| 160 | 6 | 1 | 6 | 6 | 2 |
| 161 | 4 | 2 | 6 | 6 | 4 |
| 162 | 4 | 2 | 7 | 4 | 5 |
| 163 | 4 | 1 | 1 | 4 | 4 |
| 164 | 7 | 2 | 6 | 7 | 3 |
| 165 | 7 | 2 | 4 | 7 | 4 |
| 166 | 6 | 6 | 6 | 6 | 5 |
| 167 | 2 | 2 | 6 | 2 | 4 |
| 168 | 4 | 1 | 1 | 6 | 4 |
| 169 | 6 | 4 | 6 | 6 | 4 |
| 170 | 6 | 2 | 4 | 2 | 2 |
| 171 | 6 | 4 | 2 | 6 | 5 |
| 172 | 7 | 1 | 1 | 7 | 1 |
| 173 | 2 | 6 | 1 | 7 | 2 |
| 174 | 7 | 2 | 7 | 7 | 3 |
| 175 | 4 | 6 | 4 | 4 | 5 |
| 176 | 4 | 1 | 4 | 6 | 4 |
| 177 | 6 | 6 | 6 | 6 | 4 |
| 178 | 7 | 7 | 7 | 6 | 4 |
| 179 | 4 | 4 | 4 | 4 | 3 |
| 180 | 1 | 2 | 4 | 7 | 4 |
| 181 | 7 | 2 | 4 | 4 | 5 |
| 182 | 6 | 2 | 4 | 2 | 2 |
| 183 | 6 | 6 | 4 | 6 | 5 |
| 184 | 7 | 1 | 7 | 7 | 1 |
| 185 | 1 | 1 | 1 | 1 | 4 |
| 186 | 4 | 1 | 7 | 7 | 4 |
| 187 | 6 | 1 | 1 | 6 | 5 |
| 188 | 7 | 4 | 6 | 7 | 3 |
| 189 | 6 | 6 | 4 | 6 | 3 |
| 190 | 6 | 2 | 7 | 6 | 3 |
| 191 | 6 | 6 | 6 | 6 | 2 |
| 192 | 6 | 6 | 2 | 2 | 3 |
| 193 | 4 | 4 | 7 | 1 | 3 |
| 194 | 4 | 4 | 4 | 4 | 1 |
| 195 | 4 | 2 | 2 | 6 | 3 |
| 196 | 6 | 2 | 6 | 7 | 1 |
| 197 | 4 | 7 | 1 | 4 | 1 |
| 198 | 4 | 4 | 4 | 4 | 1 |
| 199 | 7 | 7 | 4 | 6 | 4 |
| 200 | 4 | 6 | 4 | 6 | 2 |
| 201 | 4 | 6 | 2 | 6 | 3 |
| 202 | 4 | 1 | 6 | 7 | 5 |
| 203 | 6 | 6 | 6 | 6 | 3 |
| 204 | 1 | 1 | 1 | 1 | 3 |
| 205 | 6 | 4 | 6 | 2 | 5 |
| 206 | 7 | 7 | 7 | 7 | 3 |
| 207 | 4 | 2 | 4 | 4 | 3 |
| 208 | 4 | 2 | 4 | 4 | 3 |
| 209 | 6 | 1 | 6 | 4 | 4 |
| 210 | 6 | 2 | 6 | 6 | 4 |
| 211 | 6 | 6 | 6 | 6 | 5 |
| 212 | 4 | 2 | 4 | 4 | 5 |
| 213 | 4 | 4 | 4 | 4 | 3 |
| 214 | 6 | 4 | 2 | 6 | 3 |
| 215 | 4 | 6 | 6 | 4 | 3 |
| 216 | 6 | 2 | 6 | 6 | 3 |
| 217 | 4 | 4 | 4 | 4 | 3 |
| 218 | 2 | 2 | 2 | 2 | 4 |
| 219 | 4 | 6 | 6 | 6 | 1 |
| 220 | 7 | 6 | 1 | 7 | 3 |
| 221 | 4 | 4 | 4 | 4 | 2 |
| 222 | 7 | 2 | 6 | 6 | 3 |
| 223 | 6 | 4 | 4 | 6 | 1 |
| 224 | 2 | 4 | 4 | 4 | 5 |
| 225 | 6 | 6 | 4 | 6 | 5 |
| 226 | 4 | 6 | 4 | 4 | 1 |
| 227 | 4 | 4 | 4 | 4 | 2 |
| 228 | 4 | 4 | 1 | 6 | 4 |
| 229 | 4 | 1 | 6 | 4 | 5 |
| 230 | 7 | 1 | 7 | 7 | 3 |
| 231 | 6 | 6 | 6 | 6 | 2 |
| 232 | 4 | 6 | 4 | 4 | 5 |
| 233 | 4 | 2 | 6 | 7 | 4 |
| 234 | 6 | 2 | 6 | 6 | 3 |
| 235 | 4 | 4 | 4 | 4 | 1 |
| 236 | 7 | 7 | 6 | 7 | 2 |
| 237 | 7 | 7 | 1 | 7 | 4 |
| 238 | 1 | 1 | 1 | 6 | 4 |
| 239 | 2 | 4 | 2 | 1 | 2 |
| 240 | 7 | 2 | 6 | 7 | 4 |
| 241 | 4 | 2 | 7 | 6 | 2 |
| 242 | 6 | 4 | 4 | 6 | 3 |
| 243 | 4 | 4 | 4 | 4 | 5 |
| 244 | 6 | 4 | 7 | 6 | 5 |
| 245 | 4 | 1 | 7 | 6 | 4 |
| 246 | 7 | 6 | 6 | 7 | 4 |
| 247 | 4 | 2 | 7 | 7 | 5 |
| 248 | 4 | 2 | 1 | 7 | 4 |
| 249 | 6 | 1 | 4 | 7 | 4 |
| 250 | 4 | 4 | 1 | 4 | 1 |
| 251 | 4 | 7 | 2 | 4 | 3 |
| 252 | 6 | 7 | 4 | 6 | 5 |
| 253 | 4 | 1 | 1 | 6 | 5 |
| 254 | 1 | 1 | 1 | 1 | 3 |
| 255 | 6 | 4 | 2 | 6 | 4 |
| 256 | 4 | 4 | 6 | 6 | 4 |
| 257 | 6 | 2 | 2 | 6 | 5 |
| 258 | 4 | 1 | 1 | 4 | 5 |
| 259 | 7 | 7 | 6 | 6 | 2 |
| 260 | 7 | 6 | 4 | 7 | 1 |
| 261 | 7 | 7 | 1 | 6 | 4 |
| 262 | 7 | 1 | 6 | 7 | 4 |
| 263 | 6 | 4 | 6 | 2 | 3 |
| 264 | 4 | 4 | 6 | 6 | 4 |
| 265 | 7 | 1 | 7 | 7 | 2 |
| 266 | 6 | 4 | 4 | 6 | 1 |
| 267 | 4 | 4 | 4 | 4 | 4 |
| 268 | 4 | 2 | 2 | 4 | 3 |
| 269 | 6 | 4 | 2 | 4 | 4 |
| 270 | 6 | 2 | 6 | 2 | 1 |
| 271 | 6 | 2 | 6 | 6 | 2 |
| 272 | 6 | 2 | 2 | 6 | 3 |
| 273 | 4 | 4 | 1 | 4 | 4 |
| 274 | 7 | 1 | 7 | 7 | 1 |
| 275 | 4 | 1 | 4 | 6 | 4 |
| 276 | 6 | 2 | 7 | 7 | 4 |
| 277 | 4 | 2 | 4 | 4 | 5 |
| 278 | 4 | 2 | 2 | 4 | 4 |
| 279 | 6 | 1 | 4 | 6 | 4 |
| 280 | 6 | 6 | 1 | 6 | 2 |
| 281 | 6 | 2 | 1 | 6 | 4 |
| 282 | 7 | 4 | 6 | 6 | 4 |
| 283 | 6 | 2 | 4 | 6 | 1 |
| 284 | 6 | 4 | 1 | 6 | 2 |
| 285 | 4 | 6 | 6 | 6 | 4 |
| 286 | 6 | 6 | 6 | 6 | 4 |
| 287 | 6 | 2 | 6 | 6 | 4 |
| 288 | 6 | 1 | 7 | 6 | 1 |
| 289 | 2 | 7 | 6 | 4 | 2 |
| 290 | 4 | 4 | 4 | 6 | 1 |
| 291 | 7 | 1 | 6 | 7 | 2 |
| 292 | 6 | 6 | 4 | 6 | 4 |
| 293 | 7 | 6 | 4 | 7 | 2 |
| 294 | 6 | 6 | 2 | 6 | 4 |
| 295 | 6 | 6 | 6 | 6 | 4 |
| 296 | 7 | 7 | 7 | 7 | 3 |
| 297 | 1 | 4 | 1 | 6 | 1 |
| 298 | 4 | 7 | 4 | 4 | 5 |
| 299 | 4 | 6 | 1 | 2 | 3 |
| 300 | 4 | 4 | 4 | 4 | 4 |
| 301 | 7 | 1 | 7 | 7 | 5 |
| 302 | 4 | 1 | 1 | 4 | 4 |
| 303 | 6 | 2 | 6 | 6 | 4 |
| 304 | 7 | 2 | 6 | 7 | 3 |
| 305 | 4 | 6 | 6 | 6 | 2 |
| 306 | 6 | 6 | 6 | 6 | 2 |
| 307 | 4 | 4 | 2 | 4 | 4 |
| 308 | 2 | 1 | 4 | 4 | 1 |
| 309 | 4 | 4 | 1 | 6 | 4 |
| 310 | 7 | 6 | 7 | 7 | 2 |
| 311 | 4 | 4 | 4 | 6 | 1 |
| 312 | 6 | 2 | 1 | 6 | 4 |
| 313 | 4 | 4 | 6 | 6 | 1 |
| 314 | 4 | 7 | 4 | 2 | 4 |
| 315 | 7 | 6 | 1 | 7 | 4 |
| 316 | 4 | 2 | 4 | 4 | 5 |
| 317 | 4 | 1 | 6 | 7 | 3 |
| 318 | 6 | 6 | 4 | 6 | 5 |
| 319 | 7 | 4 | 2 | 7 | 4 |
| 320 | 7 | 7 | 1 | 2 | 5 |
| 321 | 6 | 1 | 1 | 4 | 3 |
| 322 | 7 | 7 | 1 | 7 | 1 |
| 323 | 7 | 2 | 7 | 7 | 4 |
| 324 | 4 | 7 | 4 | 6 | 5 |
| 325 | 7 | 1 | 7 | 7 | 4 |
| 326 | 6 | 6 | 6 | 6 | 4 |
| 327 | 2 | 2 | 6 | 2 | 3 |
| 328 | 6 | 4 | 4 | 6 | 1 |
| 329 | 1 | 1 | 1 | 1 | 4 |
| 330 |  |  |  |  | 5 |
