# Supplementary material for: Opportunities and challenges within green spaces during COVID-19: Perspectives of visitors and managers in Maine, USA
Source: PLoS One. 2025 Apr 22;20(4):e0320800. doi: 10.1371/journal.pone.0320800 (PMC12013927; doi:10.1371/journal.pone.0320800)
Supplement: Attachment — Submitted filename: Reviewer Comments - Feb2024.docx [file pone.0320800.s005.docx]

PONE-D-24-14900

Opportunities and challenges within green spaces during COVID-19: Perspectives of visitors and managers in Maine, USA

PLOS ONE

Dear Dr. Soucy,

Thank you for submitting your manuscript to PLOS ONE. After careful consideration, we feel that it has merit but does not fully meet PLOS ONE’s publication criteria as it currently stands. Therefore, we invite you to submit a revised version of the manuscript that addresses the points raised during the review process.

The third reviewer has not yet submitted their review report. However, I believe it would be beneficial to forward the current reviews to you in order to facilitate the review process. Should a late review be received, it will be sent to you.

Please submit your revised manuscript by Oct 05 2024 11:59PM. If you will need more time than this to complete your revisions, please reply to this message or contact the journal office at plosone@plos.org. When you're ready to submit your revision, log on to <https://www.editorialmanager.com/pone/> and select the 'Submissions Needing Revision' folder to locate your manuscript file.

Please include the following items when submitting your revised manuscript:

- A rebuttal letter that responds to each point raised by the academic editor and reviewer(s). You should upload this letter as a separate file labeled 'Response to Reviewers'.
- A marked-up copy of your manuscript that highlights changes made to the original version. You should upload this as a separate file labeled 'Revised Manuscript with Track Changes'.
- An unmarked version of your revised paper without tracked changes. You should upload this as a separate file labeled 'Manuscript'.

If you would like to make changes to your financial disclosure, please include your updated statement in your cover letter. Guidelines for resubmitting your figure files are available below the reviewer comments at the end of this letter.

If applicable, we recommend that you deposit your laboratory protocols in [protocols.io](http://protocols.io/) to enhance the reproducibility of your results. Protocols.io assigns your protocol its own identifier (DOI) so that it can be cited independently in the future. For instructions see: [https://journals.plos.org/plosone/s/submission-guidelines#loc-laboratory-protocols](https://c05y1x9s.r.us-east-2.awstrack.me/L0/https:%2F%2Fjournals.plos.org%2Fplosone%2Fs%2Fsubmission-guidelines%23loc-laboratory-protocols/1/010f019175a2097a-b6a86d38-af4b-4513-8d98-9845a1110aa4-000000/XASue9Rl3Nq5xMq-IGKaWinuaTg=173). Additionally, PLOS ONE offers an option for publishing peer-reviewed Lab Protocol articles, which describe protocols hosted on [protocols.io](http://protocols.io/). Read more information on sharing protocols at [https://plos.org/protocols?utm_medium=editorial-email&utm_source=authorletters&utm_campaign=protocols](https://c05y1x9s.r.us-east-2.awstrack.me/L0/https:%2F%2Fplos.org%2Fprotocols%3Futm_medium=editorial-email%26utm_source=authorletters%26utm_campaign=protocols/1/010f019175a2097a-b6a86d38-af4b-4513-8d98-9845a1110aa4-000000/a73_vxibnzctqudQCY3J7O1CJug=173).

We look forward to receiving your revised manuscript.

Kind regards,

Mario Soliño
Academic Editor
PLOS ONE

**Journal Requirements:**

**When submitting your revision, we need you to address these additional requirements.**

**1. Please ensure that your manuscript meets PLOS ONE's style requirements, including those for file naming. The PLOS ONE style templates can be found at**
[**https://journals.plos.org/plosone/s/file?id=wjVg/PLOSOne_formatting_sample_main_body.pdf**](https://journals.plos.org/plosone/s/file?id=wjVg/PLOSOne_formatting_sample_main_body.pdf) **and**
[**https://journals.plos.org/plosone/s/file?id=ba62/PLOSOne_formatting_sample_title_authors_affiliations.pdf**](https://journals.plos.org/plosone/s/file?id=ba62/PLOSOne_formatting_sample_title_authors_affiliations.pdf)

Response: Thank you for including the style guide, we have made the necessary changes to manuscript formatting to meet PLOS ONE’s style requirements.

**2. Thank you for stating the following financial disclosure:
"This work was supported by the National Science Foundation under the Grants No. 1824961, 1828466, 1849802; the USDA National Institute of Food and Agriculture, McIntire Stennis project number #ME0-42017 through the Maine Agricultural & Forest Experiment Station; and by a University of Maine Research Reinvestment Fund Rural Health and Wellbeing Grand Challenge grant."**

**Please state what role the funders took in the study. If the funders had no role, please state: ""The funders had no role in study design, data collection and analysis, decision to publish, or preparation of the manuscript.""
If this statement is not correct you must amend it as needed.
Please include this amended Role of Funder statement in your cover letter; we will change the online submission form on your behalf.**

Response: Thank you for highlighting this need, we have added the above statement to the manuscript.

**3. We note that your Data Availability Statement is currently as follows: All relevant data are within the manuscript and its Supporting Information files**

**Please confirm at this time whether or not your submission contains all raw data required to replicate the results of your study. Authors must share the “minimal data set” for their submission. PLOS defines the minimal data set to consist of the data required to replicate all study findings reported in the article, as well as related metadata and methods (**[**https://journals.plos.org/plosone/s/data-availability#loc-minimal-data-set-definition**](https://journals.plos.org/plosone/s/data-availability#loc-minimal-data-set-definition)**).**

**For example, authors should submit the following data:**

**- The values behind the means, standard deviations and other measures reported;
- The values used to build graphs;
- The points extracted from images for analysis.**

**Authors do not need to submit their entire data set if only a portion of the data was used in the reported study.**

**If your submission does not contain these data, please either upload them as Supporting Information files or deposit them to a stable, public repository and provide us with the relevant URLs, DOIs, or accession numbers. For a list of recommended repositories, please see** [**https://journals.plos.org/plosone/s/recommended-repositories**](https://journals.plos.org/plosone/s/recommended-repositories)**.**

**If there are ethical or legal restrictions on sharing a de-identified data set, please explain them in detail (e.g., data contain potentially sensitive information, data are owned by a third-party organization, etc.) and who has imposed them (e.g., an ethics committee). Please also provide contact information for a data access committee, ethics committee, or other institutional body to which data requests may be sent. If data are owned by a third party, please indicate how others may request data access.**

Response: We have included them as Supplemental Information individual files for each of the three quantitative studies containing relevant data sets.

**4. Please include your full ethics statement in the ‘Methods’ section of your manuscript file. In your statement, please include the full name of the IRB or ethics committee who approved or waived your study, as well as whether or not you obtained informed written or verbal consent. If consent was waived for your study, please include this information in your statement as well.**

Response: We have modified the ethics statement to include “The University of Maine Institutional Review Board (IRB) for research…”

**5. Please review your reference list to ensure that it is complete and correct. If you have cited papers that have been retracted, please include the rationale for doing so in the manuscript text, or remove these references and replace them with relevant current references. Any changes to the reference list should be mentioned in the rebuttal letter that accompanies your revised manuscript. If you need to cite a retracted article, indicate the article’s retracted status in the References list and also include a citation and full reference for the retraction notice.**

Response: We have checked all the references for accuracy, as well as reformatted the reference style to meet journal guidelines.

**[Note: HTML markup is below. Please do not edit.]

Reviewers' comments:

Reviewer's Responses to Questions**

**Comments to the Author

1. Is the manuscript technically sound, and do the data support the conclusions?

The manuscript must describe a technically sound piece of scientific research with data that supports the conclusions. Experiments must have been conducted rigorously, with appropriate controls, replication, and sample sizes. The conclusions must be drawn appropriately based on the data presented.**

**Reviewer #1: Yes**

**Reviewer #2: Yes**

**2. Has the statistical analysis been performed appropriately and rigorously?**

**Reviewer #1: Yes**

**Reviewer #2: N/A**

**3. Have the authors made all data underlying the findings in their manuscript fully available?

The** [**PLOS Data policy**](http://c05y1x9s.r.us-east-2.awstrack.me/L0/http:%2F%2Fwww.plosone.org%2Fstatic%2Fpolicies.action%23sharing/1/010f019175a2097a-b6a86d38-af4b-4513-8d98-9845a1110aa4-000000/E38yWoVrDhOXoKmVeoQVrt0HjQw=173) **requires authors to make all data underlying the findings described in their manuscript fully available without restriction, with rare exception (please refer to the Data Availability Statement in the manuscript PDF file). The data should be provided as part of the manuscript or its supporting information, or deposited to a public repository. For example, in addition to summary statistics, the data points behind means, medians and variance measures should be available. If there are restrictions on publicly sharing data—e.g. participant privacy or use of data from a third party—those must be specified.**

**Reviewer #1: Yes**

**Reviewer #2: Yes**

**4. Is the manuscript presented in an intelligible fashion and written in standard English?

PLOS ONE does not copyedit accepted manuscripts, so the language in submitted articles must be clear, correct, and unambiguous. Any typographical or grammatical errors should be corrected at revision, so please note any specific errors here.**

**Reviewer #1: Yes**

**Reviewer #2: Yes**

**5. Review Comments to the Author

Please use the space provided to explain your answers to the questions above. You may also include additional comments for the author, including concerns about dual publication, research ethics, or publication ethics. (Please upload your review as an attachment if it exceeds 20,000 characters)**

**Reviewer #1: The paper is well written, adequately describes methods and results, had presents implications for managers.
My only concern is that not all the concepts used in the research are fully detailed in the literature review (e.g. place attachment is a complex topic, that needs more explanation, if the results related to identity and dependence are to be useful to readers.)**

Response: Thank you for bringing up this important point regarding further elaboration of sense of place/place attachment. First, we moved the definitions of sense of place/place attachment from study site description to the section on “People and green spaces.” We further elaborated within this paragraph to offer more context in regards to how sense of place is linked with green spaces, including the following: “Sense of place significantly influences not only individuals’ experiences within green spaces, but also their trust in the management of these areas [57], motivations for recreation visits and satisfaction with their visits [58], and willingness to travel to or advocate for certain locations [59]. For example, studies have shown that strong place identity fosters trust in park authorities and stewardship, while high place dependence enhances satisfaction and loyalty to a specific green space [60,61].”

**Reviewer #2: General Comments
The paper is well-structured and well-written, focusing on the effects of the COVID-19 pandemic on green areas in Maine, USA. The study employs a qualitative methodology, comparing different projects to understand how people, due to the pandemic, changed their ways of interacting with the green areas in the case study analyzed. It aims to explore the opportunities and challenges related to changing customs in the use of green areas, particularly the influence of COVID-19 on these changes in activities, perceptions, and uses within the case study. This topic is both interesting and necessary. However, the paper could benefit from a more in-depth examination of certain aspects to enhance the robustness of the results and reinforce the research gap and the importance of conducting similar studies.**

**Below, I provide some comments and suggestions that can help to improve the paper, in case the authors want to consider them.
Specifically, I recommend that the authors expand the Introduction section to include more background on the research related to COVID-19 and the importance of green areas. Recent studies, in fact, have explored this topic from different perspectives. Additionally, it would be beneficial to clearly specify the research gap and the innovative contributions this article makes to the literature, differentiating it from other related studies. This will help to increase the robustness and novelty of the current study by clarifying its unique contributions compared to other research. Currently it is not so clear to me reading the text the novelty that the paper brings if we compare it with other literature on the same topic. The Introduction and Conclusions are likely good sections in which to highlight these aspects in more detail.**

Response: We appreciate your careful review of our manuscript, and identification of a need to further articulate the novelty of our work. Underscoring the innovativeness of our work was a key focus of the revision. First, we expanded the literature review to include more background on COVID-19 influences on recreation to provide further context on closures, restrictions, and the importance of green spaces for social interactions (see “Emergence of COVID-19 in the context of green spaces”). Additionally, we added the following paragraph at the end of the introduction to further situate our work:

“This case study provides a novel and innovative contribution to research on COVID-19 and green spaces by examining diverse perceptions and pandemic-related impacts through the lens of place attachment within a rural region. Existing studies have highlighted changes in visitation during COVID-19 and its benefits for mental and physical health; however, this research distinguishes itself by focusing on Maine, a state characterized by its expansive green spaces and its reliance on tourism. Maine offers a compelling lens for understanding how place attachment—a sense of identity, connection, and emotional bond to specific locations—influenced responses to the pandemic and patterns of use and stewardship. By incorporating diverse perspectives from green space managers and visitors, this study captures the layered meanings and values associated with these spaces during the pandemic, offering transferable insights for other rural, tourism-dependent regions. Furthermore, the study’s snapshot approach documents specific moments in time, complementing longitudinal research by revealing how place attachment evolved under crisis conditions [20]. These findings underscore the importance of integrating place-based values into adaptive management strategies to sustain green spaces into the future. This research provides a critical framework for policymakers and resource managers to balance ecological preservation with public access, while fostering meaningful connections that enhance both individual well-being and long-term conservation goals.”

**Specific comments
- Introduction – Literature Review section. In lines 92-95 and 100-102, the authors state that people have positive and negative experiences in green areas due to various factors, but do not go into detail. Can you provide examples or further elaborate on how people perceive green spaces differently according to the literature? What roles do values and socio-cultural or individual factors play in influencing these experiences?**

Response: This is an important point and therefore we added additional context to clarify on the positive and negative experiences associated with green spaces. For example, we added the following points to the first paragraph under “People and green spaces”: However, experiences, historical relationships with land (i.e., exclusion from land), sociocultural practices, family roles, sense of belonging, experiences with discrimination, and resource availability informs how groups engage with the outdoors and the types of experiences they derive [32,33]. Individuals may feel anxious or uncertain concerning the use of green spaces, which can ultimately inhibit the benefits of green spaces and make people feel unwelcomed [34,35]. In other words, the benefits of outdoor recreation have not been equitably shared across all communities [33]. Factors such as race and ethnicity, economic status, and gender have significantly impacted the extent to which structural, interpersonal, and intrapersonal barriers influence experiences in the outdoors [36–39]. For example, during COVID-19, the majority of individual those that participated in outdoor recreation in the U.S. – including those that were new to outdoor recreation – identified as being white, highlighting inequities in outdoor recreation and access [39].

**- Line 132-135. Can you further explain in the text what causes an increase or decrease? What are the most common factors you found in the literature?**

Response: We have added the following to this paragraph: **“**[66]. Closures in particular were significantly detrimental to parks globally as they are reliant on tourism for financial stability [4]. On a national scale, restrictions varied based on location, and visitors responded in kind — in Maine for example, stricter pandemic protocols caused a 27% decline in visitation from 2019 to 2020 while states with fewer pandemic restrictions such as New Hampshire experienced an influx of often non-compliant visitors and over-congestion [24]. The high demand for outdoor recreation was due in part to the perceived safety that socially engaging in the outdoors presented in comparison to indoor gatherings, a sentiment that was encouraged by many state and local governments, and the lack of indoor gathering opportunities at the beginning of the pandemic [2,4,64,67].”

**- Line 178. Figure 1. I think adding a figure of the green areas in the case study analyzed, with their distribution, different typologies, or some representative photos, could help the reader better understand the context being analyzed and the results obtained later.**

Response: This is a great idea; unfortunately, given that visitors recreated throughout the state (with the exception of Acadia specific information) and the specific locations of managers in the QM-21 and IM-21 studies is confidential, it is not feasible to include representative photos.

**- Line 186. Are you referring to visitors, local citizens, or tourists? Is the sense of place the same for people who don't live in the area compared to local people? Are you considering these distinctions, or are you talking about all types of visitors in these green spaces? It would be better to specify this in the text.**

Response: We have specified that we are referring to both local and out-of-state visitors.

**- Line 234. Can you add more information on how you triangulated the participants and methods? This could help increase the ease for readers who want to replicate the same methodology.**

Response: Thank you for highlighting this need for further elaboration! We have added the following the “Description of the overarching case study methodology,” “We simultaneously identified the common variables and constructs for comparison across quantitative studies, while exploring the relevant COVID-19 related themes in the qualitative studies. Through in-depth team discussions, data analysis, and data visualizations (e.g., word clouds, tables, and graphs) we triangulated across participants and methods (De Urioste-Stone et al., 2018; Baxter and Jack, 2008) to understand areas of convergence and divergence. We focused on the key survey constructs which helped to illuminate the impact of COVID-19 on green space experiences and associated qualitative concepts that emerged to co-identify three key themes that all studies could engage with.”

**- Line 285-289. Can you provide further details about how you identified this 3 main category and what criteria helped you define them?**

Response: This is an important point, thank you for bringing up this question. In response, we have added the following clarification to the results section: “We conducted several cycles of analysis across all studies, comparing the similarities and differences in the data across perspectives and changes through time. We identified three themes, which focus on the categories that overlapped across all studies, and illustrate the diversity of experiences and responses: (1) Altered visitation patterns…” Further details regarding the process of triangulation (including the thought process behind criteria for theme selection) is described in the above point.

**- Line 328-329. What type of “ecological damage” or “ecological worries” are you referring to?**

Response: To further clarify, we have modified to the following: “need to minimize ecological damage (i.e., vandalizing, littering).” We have also added the following background in the section “Emergence of COVID-19 in the context of green spaces” to further situate our findings in the literature, “In national parks, reduced park staff and increased visitation during COVID-19 led to increases in poaching and illegal activities (i.e., littering, unregulated camping/hiking/fishing, and public urination/defecation due to reduced visitor monitoring capacity and access to public facilities) (Jacobs et al., 2021; Miller-Rushing et al., 2021; Spenceley et al., 2021). Increased visitation also raised concerned regarding trampling of sensitive flora or widening of trails as individuals adhere to social distancing measures – all of which can result in ecological damage (Miller-Rushing et al., 2021).”

**- Line 402-404. Can you further elaborate on the concept of "attachment to the state"? What are you referring to? I think it can help the reader to better understand the concept of sense of place, identity, and attachment of you case study, as well as helping to extrapolate it more generally.**

Response: Thank you for alerting us to the confusing phrasing, we have modified the phrase to “place attachment to Maine” for clarity.

**- Line 414. Table 3. It seems that the terms "Place Identity" and "Place Dependence" appear here with great importance, but their meanings have not been explained in detail in the text. I think lines 187-189 would be a good place to add a paragraph briefly explaining how the authors understand these two concepts within the context of their study, as these terms can have completely different meanings depending on the socio-cultural factors of the context being analyzed.**

Response: This is an excellent point, and has been a primary focus of the revision. First, we moved the definitions of sense of place/place attachment from study site description to the section on “People and green spaces.” We further elaborated within this paragraph to offer more context in regards to how sense of place is linked with green spaces, starting with: “Sense of place significantly influences not only individuals’ experiences within green spaces, but also their trust in the management of these areas…” See also Reviewer #1 comment above.

**- Line 439-440. This reflection and suggestion is valid for all the results and discussions presented in the article. Is this perception different compared to the pre-COVID period? How was the general sense of attachment of people in your case study before the COVID-19 pandemic? Are there any differences? Could you further elaborate on this comparison between pre- and post-pandemic? How has the sense of place and identity of people changed? I think that giving more details could help to underline the innovative insights that have come to light with this study and give it greater robustness**

Response: This is a great question and further underscores the importance of place attachment to visitation within Maine’s green spaces. We have added the following to the discussion to further draw comparisons to previous work, “Research prior to COVID-19 suggests that visitors’ place attachment in Maine was relatively lower in 2016 compared to the pandemic and post-pandemic [De Urioste-Stone, personal communication]. The COVID-19 pandemic therefore may have brought visitors with higher place attachments and/or bolstered place attachment for return visitors. As a result, supporting Maine’s ability to draw in visitors.” Specifically, we have access to unpublished data from a 2016 study where place identity and place dependence were lower for Maine visitors.

**- Line 591-593. You should discuss more about the values, norms, and perceptions of the people, as individual and community factors, in the case study analyzed, both as individual and community factors, in the case study analyzed, not just referring to other literature. Could you provide some concrete examples related to your results? This will strengthen the connection between results and discussion sections.**

Response: We agree that this would be something relevant to add, but unfortunately understanding the values and norms of visitors was out of the scope of the quantitative studies within the case study. We have added the following to the conclusions: “In addition, further work could explore more in-depth the values and norms of Maine visitors, building on the quantitative analysis in this study.”

**- Line 721. Could you further elaborate on the importance of local values and socio-cultural factors in influencing people's changing attitudes and perceptions regarding the use of green areas? How can these factors could influence the results?**

Response: Thank you for bringing up this need to further elaborate – local values, perceptions, input, etc. are important within green space decision-making. We have added the following to this section to clarify, “Therefore, future work would benefit from an inclusion of local residents – a group we did not account for in this case study and who would be directly impacted by green space management decisions, and visitation impacts. Local residents also have the ability to influence green space management via public input (i.e., supporting or opposing visitation restrictions), and therefore represent a missing actor in the study.”

**- In the conclusions section, I would recommend that the authors to further emphasize the novelty of their study, distinguishing it from other literature that studies similar aspects related to COVID-19 and the change in people's perceptions and attitudes towards green areas.**

Response: This is a much appreciated point, and we have added the following to the conclusion to further highlight the novelty of this case study, “Maine provided a unique case study due to its popularity as a vacation destination for these metropolitan areas and possession of the only national park on the U.S. East Coast. In addition, Maine is an interesting case study given that visitors had a relatively high place attachment to the state as compared to pre-pandemic visitors. Therefore, the state’s green spaces continued to see visitation, causing opportunities and challenges for managers already burdened by staff and resource restrictions. Over the course of the pandemic, a heightened sense of place...”

**6. PLOS authors have the option to publish the peer review history of their article (**[**what does this mean?**](https://c05y1x9s.r.us-east-2.awstrack.me/L0/https:%2F%2Fjournals.plos.org%2Fplosone%2Fs%2Feditorial-and-peer-review-process%23loc-peer-review-history/1/010f019175a2097a-b6a86d38-af4b-4513-8d98-9845a1110aa4-000000/W2SnuTFGM68aELE4O1dF0q_fwoY=173)**). If published, this will include your full peer review and any attached files.


If you choose “no”, your identity will remain anonymous but your review may still be made public.
Do you want your identity to be public for this peer review? For information about this choice, including consent withdrawal, please see our** [**Privacy Policy**](https://c05y1x9s.r.us-east-2.awstrack.me/L0/https:%2F%2Fwww.plos.org%2Fprivacy-policy/1/010f019175a2097a-b6a86d38-af4b-4513-8d98-9845a1110aa4-000000/pESd0TaSlSeThP7MhfLfJlh8-cY=173)**.**

**Reviewer #1: No**

**Reviewer #2: Yes: Enrica Garau**
